# The combined diabetes and renal control trial (C-DIRECT) - a feasibility randomised controlled trial to evaluate outcomes in multi-morbid patients with diabetes and on dialysis using a mixed methods approach

**DOI:** 10.1186/s12882-018-1183-z

**Published:** 2019-01-03

**Authors:** K. Griva, M. Rajeswari, M. Nandakumar, E. Y. H. Khoo, V. Y. W. Lee, C. G. Chua, Z. S. Goh, Y. T. D. Choong, S. P. Newman

**Affiliations:** 1 Centre for Population Health Sciences, Clinical Sciences Building, Lee Kong Chian School of Medicine, Nanyang Technological University Singapore, Singapore, 308232 Singapore; 2 Department of Psychology, National University of Singapore, 9 Arts Link AS4, Singapore, 117570 Singapore; 3 National Kidney Foundation, 81 Kim Keat Road, Singapore, 328836 Singapore; 4 Department of Medicine, Yong Loo Lin School of Medicine, National University Singapore, Singapore, Singapore; 5 Division of Endocrinology, University Medicine Cluster, National University Health System, Singapore, Singapore; 6 School of Health Sciences, City University of London, Northampton Square, London, UK

**Keywords:** Self-management, Diabetes, Kidney disease, End stage renal disease

## Abstract

**Background:**

This cluster randomised controlled trial set out to investigate the feasibility and acceptability of the “Combined Diabetes and Renal Control Trial” (C-DIRECT) intervention, a nurse-led intervention based on motivational interviewing and self-management in patients with coexisting end stage renal diseases and diabetes mellitus (DM ESRD). Its efficacy to improve glycaemic control, as well as psychosocial and self-care outcomes were also evaluated as secondary outcomes.

**Methods:**

An assessor-blinded, clustered randomised-controlled trial was conducted with 44 haemodialysis patients with DM ESRD and ≥ 8% glycated haemoglobin (HbA1c), in dialysis centres across Singapore. Patients were randomised according to dialysis shifts. 20 patients were assigned to intervention and 24 were in usual care. The C-DIRECT intervention consisted of three weekly chair-side sessions delivered by diabetes specialist nurses. Data on recruitment, randomisation, and retention, and secondary outcomes such as clinical endpoints, emotional distress, adherence, and self-management skills measures were obtained at baseline and at 12 weeks follow-up. A qualitative evaluation using interviews was conducted at the end of the trial.

**Results:**

Of the 44 recruited at baseline, 42 patients were evaluated at follow-up. One patient died, and one discontinued the study due to deteriorating health. Recruitment, retention, and acceptability rates of C-DIRECT were generally satisfactory HbA1c levels decreased in both groups, but C-DIRECT had more participants with HbA1c < 8% at follow up compared to usual care. Significant improvements in role limitations due to physical health were noted for C-DIRECT whereas levels remained stable in usual care. No statistically significant differences between groups were observed for other clinical markers and other patient-reported outcomes. There were no adverse effects.

**Conclusions:**

The trial demonstrated satisfactory feasibility. A brief intervention delivered on bedside as part of routine dialysis care showed some benefits in glycaemic control and on QOL domain compared with usual care, although no effect was observed in other secondary outcomes. Further research is needed to design and assess interventions to promote diabetes self-management in socially vulnerable patients.

**Trial registration number:**

Trial registered with the International Standard Randomised Controlled Trial (ISRCTN10546597). Registered 12 September 2016 (Retrospectively registered).

## Background

Healthcare services worldwide are placing increasing emphasis on patient activation towards health. Improved self-management has been identified as key in improving disease outcomes and quality of life for people with long-term conditions and relevant interventions have the potential to yield benefits for patients and health care systems alike [1].

Managing treatment and self-care is a challenge for patients with chronic multi-morbidity. Comorbid diabetes mellitus and end-stage renal disease (DM ESRD) is a major and emerging condition that health care services must manage. Diabetic nephropathy is the leading cause of ESRD, with as many as 50% of patients on renal replacement therapy being diagnosed with DM ESRD [2, 3]. With the diabetes epidemic and the ageing populations, these rates are projected to rise further. DM ESRD often leads to manifold negative health effects [4]. More than 50% of DM ESRD patients die within 2 years of dialysis initiation [5, 6], largely due to cardiovascular complications [7], and hospital admissions for DM ESRD patients exceed others by 26% [6]. Quality of life (QOL) is reportedly worse for DM ESRD patients [4, 8, 9]. Self-management and adherence are particularly problematic as regimen complexity demands intensify and may compete with each other [10, 11], making it harder for patients to integrate guidelines or decide on priorities regarding their care [12]. In addition to dialysis, the key to the management of DM ESRD lies in both medication adherence and diet modification. DM ESRD patients often face difficulties in glycaemic and phosphate control, which cannot be completely addressed with medication alone without dietary modifications. Diet is particularly challenging for DM ESRD patients as they have to abide to often complex and incompatible dietary recommendations leading to a suboptimal self-care and management [13]. Notably, DM ESRD patients are known to have “poor” records of adherence and self-management. Motivation to self-care may thus be undermined.

There is extensive literature on value of disease-specific programs based on self-management principles to support behavioural changes especially in the context of diabetes [14], and systematic reviews and meta-analyses have shown benefits in both glycaemic control and self-care over usual care [15–18]. Based on such strong evidence, the American Diabetes Association, the American Association of Diabetes Educators, and the Academy of Nutrition and Dietetics released a joint statement identifying self-management support as essential for all individuals with diabetes [19]. Evidence on multi-morbid patients however remains scarce [20], more so for the ESRD population [21]. Interventions to support this high-risk segment of dialysis patients are clearly needed – especially inexpensive and low intensity programs that can be delivered as part of routine care and hence have the potential to become available/accessible to more patients. A self-management program (HEDSMART) developed specifically for patients on haemodialysis (HD) has shown improved clinical and behavioural outcomes and reductions in depression [21, 22], but proportion of patients with diabetes recruited was low. Leveraging on this approach and following formative work with DM ESRD patients [23], we have developed a brief-clinic integrated intervention for patients with coexisting DM and ESRD currently treated with HD, the “Combined Diabetes and Renal Control Trial” (C-DIRECT) to support self-management for both conditions.

To gauge its potential benefit(s) and evaluate the possibility of any design issue(s) in the protocol, [24] we had to conduct a trial to establish the acceptability and feasibility of the C-DIRECT. Feasibility trials are recommended before investing in a full scale, costly trial, and even before considering for use in clinical practice. The information from this trial will be used to guide the refinement of a future adequately powered trial to evaluate the effectiveness of the program for the self-management of coexisting diabetes and ESRD.

The objective of this trial was to assess the feasibility and acceptability of C-DIRECT. We aimed to collect HbA1c and patient-reported outcomes to assess the potential efficacy of C-DIRECT to improve glycaemic control, psychosocial, and self-care outcomes of patients with coexisting DM ESRD compared with usual care so as to determine the most appropriate main outcome for the main trial.

## Method

### Trial design

This feasibility study is a parallel two-arm, assessor-blinded, clustered randomised control trial (RCT). The trial had a 1:1 allocation ratio across study arms and used a mixed methods approach for evaluation of outcomes.

Ethical approval has been granted by the National University of Singapore Institutional Review Board (NUS IRB 13–295) and the trial has been registered in the ISRTCN Registry (ISRCTN10546597).

### Setting and participants

Patients with DM ESRD who were undergoing haemodialysis were recruited from participating dialysis centres of the National Kidney Foundation of Singapore (NKF). NKF is a not-for-profit organisation that provides dialysis for lower and middle-income ESRD patients in Singapore across dialysis centres located within communities island wide. Patients admitted onto program get allocated to neighbourhood dialysis centres nearest to their residence.

Patients were eligible for the study if they were older than 21 years of age (the legal cut off for the definition of adults in Singapore), had coexisting DM and ESRD, were on HD for a minimum of 3 months, had a HbA1c level ≥ 8% (at baseline), and were able to communicate in English or Mandarin.

Exclusion criteria included the following: having a serious physical or mental health conditions that would prevent or hinder study participation (consent and assessments), being hospitalised, and fluent only in Malay, Tamil or other dialects. Criteria were assessed by senior nurse managers and using data for medical records. List of eligible patients and preferred language of communication for those patients were provided prior to data collection.

### Sample size

The sample sizes of 30 to 50 have been recommended for feasibility or pilot studies [25]. Consistent with the recommendation, we sought to recruit approximately 45 eligible participants with an estimated attrition of 10% (about 20 patients per arm).

### Randomisation

Dialysis shifts (Dialysis Shift 1: Monday/ Wednesday/ Friday; Shift 2: Tuesday/ Thursday/ Saturday), instead of individual participants, were the preferred unit of randomisation to avoid treatment contamination across groups. Randomizing by shifts rather than dialysis centres can also potentially control for differences across centres (number of beds; layout of space, etc) that may introduce unforeseen bias across study arms. Block Randomisation was undertaken following baseline assessment, using a random number sequence produced by a computer-generated program (randomizer.org).

Allocation concealment was undertaken such that the managers of the dialysis centres were not aware of the study arm allocation. The C-DIRECT intervention is delivered as part of usual patient care by the DM Link nurses. Assessors that collected and analysed the data were independent to study care team and remained blind to study arm allocation but use of study codes. It was not possible to blind facilitators and patients as the intervention required input by these parties.

### Intervention

#### Control group (usual care)

Participants in the control group received standard/ usual care for the duration of the study.

Usual care comprises regular blood tests and medical follow ups and care by multidisciplinary team including renal doctors, nurses, dietitians, exercise specialists, and medical social workers. Specialist DM link nurses support diabetes care for DM ESRD. DM link nurses are specialised renal and diabetes educators and are assigned to cover several dialysis centres in geographical clusters across the country (i.e. dialysis centres in the central; north; west and east parts of Singapore).

#### Intervention group (C-DIRECT with usual care)

Participants allocated to C-DIRECT received usual care as described above plus the intervention (C-DIRECT). The theoretical framework of the C-DIRECT was based on Social Cognition Theory and Motivational Interviewing (MI), aiming to empower patients to problem solve, set goals and stimulate valued behavioural change. The C-DIRECT intervention was modelled on the Haemodialysis Self-management Intervention Randomised Trial (HED-SMART) renal programme [26], but its content and delivery was tailored to the needs and context of coexisting DM ESRD, as identified in a previous mixed methods study [27]. These sessions were delivered at bedside (typically the first 30–60 min upon cannulation and connection to the HD machine) by DM link nurses.

The facilitators completed a 2-day group training course to deliver the C-DIRECT intervention followed by 2 sessions of one-to-one coaching. The training course consisted of 1-day on self-management principles (e.g. problem-solving; goal-setting) and 1-day on motivational interviewing (key focus on elicit-provide-elicit framework in reference to providing clinical feedback and advice). A MINT (Motivational Interviewing Network of Trainers) trainer observed facilitators for 2 pilot intervention sessions and coached/provided feedback as needed to ensure consistency of delivery and fidelity to behavioural change principles (as noted above). Arrangements for additional supervision during the trial were only on need basis or as requested by intervention facilitators.

The purpose of the sessions with patients were (a) to explore and expand understanding and activation around self-management behaviours and (b) to collaboratively set goals to work towards more effective disease management. The intervention employs the MI framework whereby key communication microskills, i.e. of open-ended questions, affirmations, reflections and summaries were used to guide the intervention sessions through the processes of engagement, focusing (target as chosen by patients), evocation, and planning [28]. The Elicit-Provide-Elicit framework was used to explore understanding and tailor advice/education on topics as well as the communication of clinical feedback on lab tests and food records. Self-management strategies included goal setting, action planning, self-monitoring, and feedback (using Elicit-Provide-Elicit framework).

At each session, facilitators presented patients with an agenda mapping chart on possible topics of discussion and patients were invited to choose their target (i.e. focusing) for the session: i.e. diet; foot care; fluid control; medications; blood glucose monitoring; monitoring (eyes); or others (to invite patients to voice other concerns/topics that would like to address in relation to management of DM ESRD).

In an integrated self-management approach, sessions were concluded with the formulation of goal-directed action plan related to patients’ chosen topic for each session. All goals were set either collaboratively with the patients or by patients themselves.

A brief outline of individual session is provided below:

Session 1:

*Provide clinical feedback on recent HbA1c Lab results using the Elicit-Provide-Elicit framework; agenda mapping (as above); provide information/advice on chosen topic using the Elicit-Provide-Elicit framework; goal setting (using importance and confidence rulers to tailor goals and behavioural contract sheet to consolidate implementation of behavioural goal) and food record (3 day) assignment.*

Session 2:

*Review goal setting progress and problem solve barriers (if any); revise goal set at session 1 as needed; review and debrief food record using the Elicit-Provide Elicit framework to provide dietary advice and support goal setting or any behavioural changes. If no issues with dietary management, agenda mapping (as above); provide information/advice on chosen topic using the elicit Provide-Elicit framework; goal setting to add new patient-initiated goal related to topic in session 2 (using importance and confidence rulers to tailor goals and behaviour).*

Session 3:

*Review goal setting progress and problems solve barriers (if any) for goal(s) set in session 2; revise goals as needed. Use Elicit-Provide-Elicit framework to problem solve lapses and barriers. Use agenda mapping (as above) to address any pending important concerns; provide information/advice on chosen topic using the elicit Provide-Elicit framework; goal setting (using importance and confidence rulers to tailor goals and behaviour) – conclude with E-P-E framework to provide additional advice and links to available resources as patients continue to move forward with their goals.*

### Outcomes

As this was a feasibility trial designed to inform future trials, several types of primary and secondary outcomes were included. These were assessed at baseline (2 weeks before randomisation) and at follow up (12 weeks post-baseline) by research coordinators who were non-interventionists and independent to the patients’ care team.

#### Primary outcomes

The primary outcomes were feasibility of recruitment, retention and acceptability of intervention. Feasibility was measured by the success of recruitment (% of consent to study; % of decline) and randomisation (% of consent to randomised; % drop out following randomisation). Recruitment and randomisation rates at ≥75% were deemed satisfactory.

Retention was measured based on attendance of sessions, ability to gather data at the time points, whether questionnaires were completed (% completion /missing data), and delivery of the intervention. Retention rates at ≥75% follow-up rate was deemed satisfactory.

The acceptability of the intervention was measured by drop-out rates and qualitative interviews conducted at the end of the trial.

#### Secondary outcomes

Secondary outcomes were the patient’s various clinical endpoints, and patient reported outcomes (i.e. quality of life, distress, adherence, and self-management skills).

##### Clinical endpoints

Data on HbA1c, biochemical markers (phosphate and potassium levels); protein catabolic rate, albumin, haemoglobin and interdialytic weight gains were collected from the study participants before the C-DIRECT, and at the end of follow up. Participants need not undergo additional blood tests as results from routine blood tests at dialysis centres were used for evaluation. Absolute levels and values within clinical target ranges were recorded.

##### Patient reported outcomes

The following standardized and psychometrically sound patient-reported measures were administered to measure distress (Problem Areas in Diabetes Scale - PAID and Hospital Anxiety and Depression Scale – HADS), quality of life (Kidney Disease Quality of Life Short Form – KDQoL), adherence and self-care (Dialysis Diet and Fluid Non-Adherence Questionnaire – DDFQ and Summary of Diabetes Self-Care Activities – SDSCA), and self-management skills (Diabetes Self Efficacy Scale – DSES and Health Education Impact Questionnaire – HEIQ). The measures are widely used in prior research and hence allow for comparability of findings. The selection of these measures also adheres to the core outcome set guidelines [29], to allow comparing the effects of different interventions in ways that maximize power and minimize bias. Linguistically appropriate versions of the questionnaires were used, and where not available, standard forward-backward translation procedures were applied. Questionnaires were self-reported but to maximize the chance of retrieving a full data set, researchers facilitated completion of forms for patients who so requested assistance with completion, by reading out the questions and/or assisting to fill up the responses (*n* = 40).

***Problem Areas in Diabetes Scale (PAID).***

The PAID is a 20-item scale used to assess the individual’s emotional adjustment towards life with diabetes [30–32]. Responses were rated from 0 (“not a problem”) to 4 (“serious problem”), and scores are then transformed on a 0 to 100 scale. Higher scores would indicate greater levels of distress. A score of 50 and higher has been recommended as a clinical significant cut-off for diabetes-related distress [33]. The PAID was demonstrated to have strong correlations with general emotional distress, depression, diabetes self-care behaviours, and was shown to be a reliable predictor of glycaemic control.

***Hospital Anxiety and Depression Scale (HADS).***

Generic symptoms of anxiety and depression were measured using the HADS, which was designed for medically ill patients and has no somatic symptom items. [34]. The HADS consists of 2 subscales of 7 items each. Each item uses a 4-point Likert scale from 0 to 3. A score of more than 8 on each of the subscale would indicate presence of anxiety or depression symptoms, and a score of more than 16 identifies caseness for the total scale. The HADS has been used extensively with HD and DM ESRD patients [35–38], and is shown to have good psychometric properties [39].

***Kidney Disease Quality of Life Short Form (KDQoL).***

Health-related quality of life was measured using the KDQoL-SF version 1.3 [40]. Both the English and Mandarin versions have been validated in the local context [41, 42]. The KDQoL-SF comprises a generic QOL portion and 43 items measuring kidney disease specific QOL. The generic QOL portion comprises 8 subscales: [1] physical functioning; [2] role physical; [3] pain; [4] general health; [5] emotional well-being; [6] role emotional; [7] social function; and [8] energy. These are combined to derive the mental component summary (MCS), physical component summary (PCS).

The kidney disease specific portion comprises nine subscales: [1] burden of kidney disease; [2] cognitive function; [3] dialysis staff encouragement; [4] effects of kidney disease; [5] patient satisfaction; [6] quality of social interaction; [7] sleep; [8] social support; and [9] symptom problem. A kidney disease component summary (KDCS) score was also calculated. All scores range from 0 to 100, with higher scores indicating better QoL.

***Dialysis Diet and Fluid Non-Adherence Questionnaire (DDFQ).***

The DDFQ measures the frequency and degree of non-adherence to diet and fluid guidelines [43]. Patients report the number of days they were non-adherent to diet and food in the past 14 days, which provides a score for frequency, and will also rate their degree (from 0 - “none” to 4 - “very severe”) in which they deviate from their guidelines, which will indicate a severity score. Lower scores in these domains would suggest lower non-adherence. The DDFQ has been found to have high criterion and construct validity.

***Summary of Diabetes Self-Care Activities (SDSCA).***

The SDSCA is a well-validated measure of diabetes self-care behaviours [44]. Its six subscales assess different domains of diabetes self-care behaviours, including general diet, specific diet, exercise, blood glucose testing, foot care and smoking. Better diabetes self-management was indicated by a higher total and subscale scores of the average number of days in a week the participant performs each self-care activity. The SDSCA has been validated on diabetic ESRD patients [23], and with other diabetic populations in Singapore [45, 46].

***Diabetes Self Efficacy Scale (DSES).***

The DSES is an eight-item scale measuring patients’ confidence about performing diabetes self-management tasks [47, 48]. The patients rate their confidence in these tasks from a scale of 0 (“not at all confident”) to 10 (“totally confident”). A higher score indicates higher self-efficacy. The scale has been widely used in patients with diabetes [49, 50], and in one study in patients with comorbid diabetes and ESRD [51].

***Health Education Impact Questionnaire (HEIQ).***

The HEIQ is a psychometric tool to evaluate patient reported outcomes of education and self-management interventions for patients with chronic conditions [52]. There are eight domains in the HEIQ: [1] positive and active engagement in life, [2] health directed behaviour, [3] skills and techniques acquisition, [4] constructive attitudes and approaches, [5] self-monitoring and insight, [6] health service navigation, [7] social integration and support, and [8] emotional well-being, with a total of 40 questions. Participants respond to each statement by marking on a 4-point Likert scale (from 1 - “Strongly Disagree” to 4 - “Strongly Agree”). Higher scores indicate better outcomes. The HEIQ has been used for the assessment of self-management programmes relating to chronic kidney disease [26, 53] and is shown to have high construct validity and internal validity (α ranging from 0.70 to 0.89).

##### Patient’s socio-demographic factors and characteristics

Participants provided socio-demographic (age, gender, ethnicity, educations, income, relationship and employment status, living arrangements) and disease-related information (age at diagnosis, treatment modality, and diabetes complications status) at baseline. In addition, medical records were reviewed to extract information on diagnosis, duration of renal replacement therapy, medication and presence of other medical conditions. The Charlson Comorbidity Index (CCI) was calculated to estimate the comorbidity burden [54]. The CCI has been found to be highly predictive of one-year mortality of patients suffering from multi-morbid illnesses [55].

##### Qualitative interviews

To complement the quantitative evaluation and further explore the acceptability of CDIRECT, in line with current MRC process evaluation guidelines [56], qualitative interviews with intervention participants and interventionists were conducted at the conclusion of the trial. An interview guide was used to maintain focus on experience with program (i.e. content, delivery and challenges), any benefit/gains gained, point of improvements, and discussion of factors that might impact efficacy. Interviews were audio recorded and transcribed verbatim. Any identifiable information was anonymized.

### Statistical methods

Statistical analyses were carried out using SPSS (Statistical Package for the Social Sciences). The potential effects were assessed with intention to treat (ITT) analysis by the baseline carried forward method [57]. Complete case analyses were conducted as sensitivity analysis. Covariance analysis was conducted to assess any between-group differences in the outcomes at follow-up, controlling for ethnicity at baseline. We added ethnicity as a covariate as this differed significantly between groups (see below).

As the study was exploratory and not purposefully powered, the derived mean differences of the subscales are reported so as to help assess the direction, and magnitude of potential effects. The proportions of values within clinical ranges (for the biomarkers and HbA1c) were evaluated using chi square and Wilcoxon test.

Thematic analysis [58] was used to analyse transcripts of the qualitative interviews in an iterative process, re-examining the earlier transcripts with the new codes derived from each round of analysis until saturation was reached. To enhance reliability, 20% of the transcripts were systematically coded independently by the first author (KG). Inter-coder reliability was excellent (pooled Kappa .92).

## Results

### Primary outcomes

#### Recruitment

**Fig. 1:**
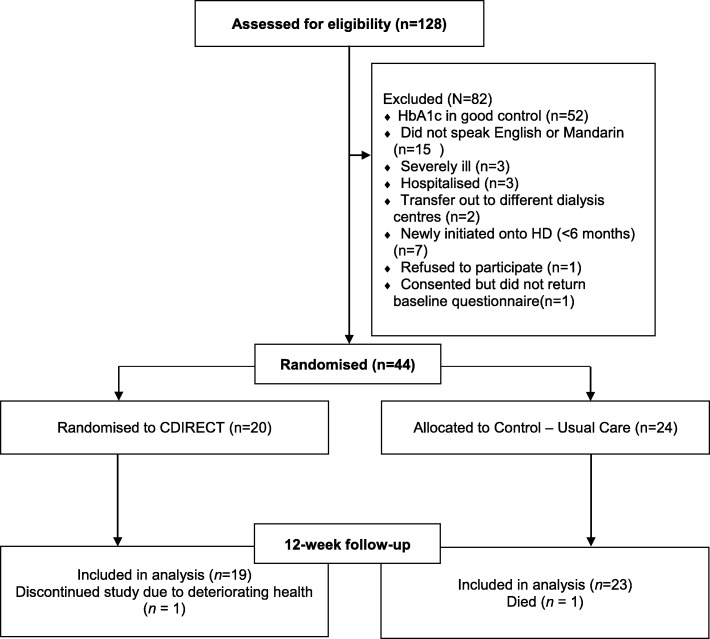
CONSORT Diagram

We screened 128 DM ESRD patients for eligibility (see Fig. 1 for CONSORT diagram). Out of them, 46 were eligible (36%); reasons for ineligibility were: HbA1c in good control (*N* = 52), did not speak English nor Mandarin (*N* = 15), severely ill (*N* = 3) or hospitalised (N = 3), transferred out to different dialysis centre (*N* = 2), or newly-initiated onto HD (less than 6 months) (*N* = 7). Of these 46 eligible patients, 1 refused to participate, and 1 consented but did not return the baseline questionnaire, resulting in 44 enrolled patients (95.7% of eligible patients enrolled) that were randomised to *N* = 20 in C-DIRECT and *N* = 24 in usual care.

None of the participants withdrew after group allocation, indicating a good level of acceptability of the randomization process.

Demographic and treatment characteristics of the trial participants are presented in Table 1. There were no significant differences between the two groups of participants. Despite randomisation, intervention group consisted of more Chinese patients than usual care (χ^2^ [1]=5.65, *p* = .032), thus all subsequent statistical comparisons controlled for ethnicity. In a larger study, stratified randomisation is recommended.

**Table 1:** Patients’ Baseline Socio-demographic and Clinical Characteristics (*n* = 44)

Measure	C-DIRECT (*n* = 20)	UC (*n* = 24)	Total Sample (*N* = 44)
Age in years	61.8 ± 9.4	63.3 ± 8.0	62.6 ± 8.6
Males	12 (60%)	9 (38%)	21 (48%)
Ethnicity			
Chinese	13 (65%)	7 (29%)	20 (46%)
Malay	6 (30%)	17 (71%)	23 (52%)
Indian	1 (5%)	0	1 (2%)
Education (years)	6.7 ± 4.1	6.65 ± 2.7	6.9 ± 3.4
Income			
< $2000	12 (60%)	7 (29%)	19 (43%)
≥ $2000	6 (30%)	4 (17%)	10 (23%)
Don’t know/ do not wish to answer	2 (10%)	13 (54%)	15 (35%)
Relationship status			
Married	13 (65%)	15 (63%)	28 (64%)
Employment Status			
Employed	3 (15%)	2 (8%)	5 (12%)
Living arrangement			
1–2 room HDB^g^ flat	4 (20%)	6 (25%)	10 (23%)
3–4 room HDB^g^ flat	14 (70%)	18 (75%)	32 (73%)
5 room HDB^g^/ executive/maisonette	2 (10%)	0	2 (5%)
Current housing			
With family	17 (85%)	23 (96%)	40 (91%)
Alone/others	3 (15%)	1 (4%)	4 (9%)
Age of diagnosis for diabetes	42.1 ± 12.0^a^	44.12 ± 12.4^c^	43.19 ± 12.1^e^
Duration of diabetes (months)	19.73 ± 10.2^a^	18.38 ± 9.1^b^	19.03 ± 9.5^f^
Age of diagnosis of CKD	51.44 ± 13.42^b^	58.24 ± 10.46^d^	55.3 ± 12.15
Duration on dialysis in years	4.37 ± 3.64	4.14 ± 2.99	4.24 ± 3.26
Charlson’s Comorbidity Index (CCI)	7.5 ± 1.40	7.13 ± 1.23	
Required assistance with survey	17 (85%)	23 (96%)	40 (91%)

*Note:* Means are presented in *M* ± *SD*

^a^*n* = 15, ^b^*n* = 16, ^c^*n* = 17, ^d^*n* = 21, ^e^*n* = 32, ^f^*n* = 31

^g^Housing Development Board

#### Retention

All patients completed the three sessions of the program. At the conclusion of the study, the follow up questionnaire was completed by *N* = 42 (retention rate = 95.5%) of patients. One patient in the intervention discontinued the study due to health deterioration and prolonged hospitalisation and one patient in usual care died. Completion of individual measures was high (completion rate = 96%).

Interviews were completed by 17 out of 20 C-DIRECT participants and all five intervention facilitators.

#### Delivery of intervention

All participants completed all 3 sessions. In all the cases the nurse facilitators were able to deliver the intervention chairside as per protocol. The sessions were typically around 30 min (Mean = 25.5; SD = 12.1).

### Secondary outcomes

#### Clinical endpoints

Table 2 shows the clinical endpoints of the study outcome measures. Baseline observations were carried forward for missing data at follow up (ITT). Group (C-DIRECT, Usual Care [UC]) × Time (baseline, follow-up) ANCOVAs controlling for ethnicity were performed on all clinical markers.

**Table 2:** Secondary study outcome measures Clinical endpoints

	C-DIRECT			Control		
Variable	Baseline	Follow up	Mean diff	Baseline	Follow up	Mean Diff
	*M* (SD)	*M* (SD)	*M* (SD)	*M* (SD)	*M* (SD)	*M* (SD)
Potassium	4.67 (0.61)	4.67 (0.88)	0 (0.56)	4.61 (0.54)	4.80 (0.67)	0.20 (0.50)
Calcium	9.30 (0.68)	9.06 (0.63)	−0.24 (0.72)	9.48 (0.48)	9.52 (0.46)	0.04 (0.52)
Phosphate	4.50 (1.11)	4.700 (1.15)	0.20 (1.43)	4.44 (1.06)	4.32 (1.23)	−0.12 (0.95)
Haemoglobin	11.29 (1.37)	11.25 (1.31)	−0.04 (1.42)	11.56 (1.24)	11.38 (0.92)	−0.19 (0.81)
HbA1c (%)	9.57 (1.37)	8.78 (1.54)	−0.80 (1.24)*	9.62 (1.40)	9.50 (2.05)	−0.13 (1.10)*
Albumin	39.00 (2.75)	38.75 (2.79)	−0.25 (1.80)	37.75 (2.75)	37.83 (2.91)	0.08 (1.67)
Interdialytic Weight Gain	3.71 (1.24)	3.41 (1.43)	−0.30 (1.01)	3.43 (1.77)	3.52 (1.48)	0.09 (1.46)

*significant main effect of time observed

There were no significant interaction effects in any of the clinical markers suggesting no differential course across study arms (Table 2).

ITT analyses indicated significant main effects for time in HbA1c (*F* [1, 40]=6.51, *p =* .02) – with mean levels across both arms significantly reduced from baseline to follow up.

The proportion of patients within clinical targets for HbA1c significantly increased at follow up for C-DIRECT from 0% at baseline to *N* = 8 (40%) at follow up (*p* = .008) where in UC only *N* = 4 (17%) has HbA1c below 8% (*p* = .12).

Per protocol (PP) analyses indicated similar patterns of results.

#### Patient reported outcomes

Table 3 depicts patient reported outcomes at baseline and at follow up. When the recommended cut-offs were applied, depressive and anxiety symptoms were in the normal range (*M*_*Depression*_ = 4.38, *SD*_*Depression*_ = 3.59 and *M*_*Anxiety*_ = 3.78, *SD*_*Anxiety*_ = 3.29), with only 18.2% and 9.1% reaching or exceeding threshold for depression or anxiety disorder (≥8) respectively. Scores on the diabetes self-efficacy scale indicated high levels of confidence in managing diabetes regimen demands, and diabetes-related distress was low (*M* = 7.54, *SD* = 1.75). Adherence and self-care were variable. In terms of diabetes self-care, adherence was greater for diet and lower for exercise.

**Table 3:** Secondary study outcome measures Patient reported outcomes

	C-DIRECT			Control		
Variable	Baseline	Follow up	Mean diff	Baseline	Follow up	Mean Diff
	*M* (SD)	*M* (SD)	*M* (SD)	*M* (SD)	*M* (SD)	*M* (SD)
Problem Areas in Diabetes Scale	28.00 (21.10)	26.00 (21.72)	−2.00 (16.65)	19.01 (14.71)	23.85 (24.34)	4.84 (20.87)
Hospital Anxiety and Depression Scale						
Anxiety	3.45 (3.98)	4.10 (3.04)	0.65 (4.12)	2.83 (3.07)	3.50 (3.35)	0.67 (2.04)
Depression	5.30 (4.12)	5.30 (4.22)	0 (3.77) ^	3.65 (2.98)	4.22 (3.88)	0.57 (3.07) ^
Kidney Disease Related Quality of Life						
Physical Functioning	32.84 (10.38)	35.00 (11.99)	2.15 (8.30)	34.63 (12.14)	37.86 (10.94)	3.22 (12.87)
Role Limitation due to Physical Health	39.90 (12.58)	48.19 (9.03)	8.29 (11.34) *^#^	44.89 (10.75)	45.85 (10.16)	0.96 (9.11) *^#^
Bodily Pain	44.70 (15.81)	46.74 (13.01)	2.04 (13.06)	48.10 (11.61)	47.25 (15.32)	0.85 (13.07)
General Health	30.73 (9.19)	33.96 (6.45)	3.14 (8.64) ^	33.69 (7.66)	34.59 (8.98)	0.90 (10.01) ^
Emotional Wellbeing	52.59 (12.10)	51.62 (13.17)	−0.98 (15.63) ^	54.79 (12.30)	49.06 (16.02)	−5.73 (15.68) ^
Role Emotional	42.66 (16.16)	44.62 (10.65)	1.96 (15.21)	50.49 (7.56)	46.29 (12.80)	−4.19 (13.22)
Social Functioning	46.97 (13.30)	47.99 (12.81)	1.01 (13.86)	52.36 (8.38)	49.84 (11.79)	−2.52 (8.02)
Energy	42.92 (10.74)	48.15 (11.82)	5.23 (15.52)	47.75 (10.35)	50.43 (13.78)	2.68 (14.76)
Physical Composite Summary	33.34 (9.95)	38.60 (8.41)	5.26 (6.58) *	33.00 (15.33)	36.47 (15.18)	3.46 (7.78) *
Mental Composite Summary	51.60 (11.87)	51.72 (12.39)	0.11 (13.19)	63.37 (31.17)	60.15 (32.13)	−3.22 (10.79)
Symptom List	75.42 (15.98)	76.82 (17.52)	1.40 (17.71)	80.82 (16.29)	79.62 (18.20)	−1.20 (17.50)
Effects of Kidney Disease	81.55 (17.90)	80.89 (18.55)	−0.65 (22.66)	87.50 (12.01)	78.57 (22.24)	−8.93 (22.98)
Burden of Kidney Disease	27.19 (20.10)	38.75 (26.02)	11.56 (27.45) *	40.36 (28.25)	45.57 (35.67)	5.21 (24.50) *
Cognitive Function	80.67 (23.68)	81.33 (20.24)	0.67 (16.32) ^	82.50 (19.24)	80.97 (26.29)	−1.53 (24.87) ^
Quality of Social Interaction	82.00 (19.36)	86.00 (19.09)	4.00 (19.75)	84.44 (25.23)	82.64 (18.88)	−1.81 (25.73)
Sleep	67.25 (17.11)	69.13 (16.21)	1.88 (19.55)	62.29 (16.33)	53.85 (22.55)	−8.44 (18.98
Social Support	80.00 (19.94)	67.50 (24.47)	−12.50 (16.99) *	81.94 (30.26)	79.86 (24.56)	− 2.08 (28.37) *
Dialysis Staff Encouragement	81.25 (25.81)	81.25 (20.88)	0 (18.14) ^	90.63 (18.15)	89.58 (15.49)	−1.04 (22.40) ^
Patient Satisfaction	60.00 (19.04)	65.00 (24.72)	5.00 (18.81)	56.25 (16.89)	63.20 (19.64)	6.95 (18.33)
Kidney Disease Component Summary	67.57 (11.54)	68.37 (14.98)	0.80 (9.30) ^	72.92 (8.81)	72.73 (12.39)	−0.18 (13.12) ^
Dialysis Diet and Fluid Non-Adherence Questionnaire						
Diet	4.65 (7.01)	10.05 (11.95)	5.40 (11.07)	4.83 (7.38)	7.58 (10.86)	2.75 (12.18)
Fluid	7.45 (11.45)	7.55 (10.12)	0.10 (7.93)	5.17 (8.18)	8.00 (10.97)	2.83 (9.80)
Summary of Diabetes Self-Care Activities Assessment						
General Diet	5.03 (2.28)	3.93 (2.84)	−1.10 (2.41)	5.17 (2.19)	4.50 (2.52)	−0.67 (2.46)
Specific Diet	4.23 (2.04)	4.43 (1.37)	0.20 (1.74)	4.52 (1.27)	4.33 (1.07)	−0.19 (1.64)
Exercise	2.55 (2.47)	3.40 (1.92)	0.85 (1.89)	2.52 (2.21)	2.85 (2.09)	0.33 (1.79)
Blood Sugar Taking	2.64 (2.51)	2.56 (2.63)	−0.08 (2.05)	2.31 (2.19)	2.98 (2.39)	0.67 (1.35)
Foot Care	2.68 (2.46)	3.26 (2.63)	0.58 (2.55)	3.10 (2.57)	3.33 (2.71)	0.23 (2.77)
Diabetes Self-Efficacy Scale Total	7.11 (1.78)	7.14 (1.67)	0.03 (1.23)	7.76 (1.71)	7.71 (1.99)	−0.06 (1.14)
Health Education Impact Questionnaire						
Positive and Active Engagement in Life	2.81 (0.49)	3.08 (0.55)	0.28 (0.51)	2.85 (0.55)	2.88 (0.70)	0.03 (0.55)
Health Directed Behaviour	2.80 (0.73)	2.97 (0.63)	0.16 (0.59)	2.84 (0.72)	2.90 (0.61)	0.05 (0.68)
Skills and Techniques Acquisition	3.03 (0.35)	3.14 (0.43)	0.11 (0.47)	2.99 (0.49)	3.12 (0.59)	0.14 (0.54)
Constructive Attitudes and Approaches	2.93 (0.54)	3.17 (0.40)	0.24 (0.62) ^	3.08 (0.50)	3.26 (0.64)	0.18 (0.48) ^
Self-Monitoring and Insight	3.16 (0.30)	3.39 (0.38)	0.23 (0.39) *	3.10 (0.28)	3.36 (0.34)	0.26 (0.35) *
Health Service Navigation	3.17 (0.40)	3.28 (0.44)	0.11 (0.52)	3.23 (0.42)	3.44 (0.48)	0.22 (0.35)
Social Integration and Support	2.83 (0.41)	3.13 (0.62)	0.30 (0.71)	3.00 (0.54)	3.18 (0.61)	0.19 (0.48)
Emotional Wellbeing	2.33 (0.73)	2.38 (0.72)	0.04 (0.95)	2.22 (0.63)	2.15 (0.74)	−0.08 (0.60)

*significant main effect of time observed

^#^significant interaction effects of time and group observed

^ significant effects of ethnicity observed

The ANCOVAS to compare study arms (C-DIRECT, Usual Care [UC]) over Time (baseline, follow-up) while controlling for ethnicity indicated only a few significant effects in patient reported outcomes.

In terms of QOL, there was significant time (*F* [1, 40]=5.17, *p =* .03) and interaction effect for role limitations due to physical health subscale of the KDQoL [*F* [1, 40]=5.44, *p* = .03)] – post hoc comparisons showed that although physical role limitation scores improved for total sample, C-DIRECT participants significantly improved more than UC in this KDQoL subscale post intervention.

Significant time effects were also noted for the following KDQoL subscales/ summary scores: PCS (F [1, 40]=11.79, *p* = .001), burden of kidney disease, (F [1, 40]=5.30, *p* = .03), and social support (F [1, 40]=5.76, *p* = .02). These indicated an increase in QOL in terms of PCS and burden but as the interaction effects were not significant, there is no support for C-DIRECT.

All other patient reported outcomes, namely adherence, diabetes self-efficacy and self-management skills (HEIQ) remained undifferentiated over time across study arms with the exception of a significant time effect for the HEIQ self-monitoring/ insight domain (F [1, 40]=15.27, *p* < .001). This indicated an increase in self-monitoring and insight skills, but since the interaction was not significant, there is no support for C-DIRECT.

It is of note, however, that both ITT and per protocol analyses showed that ethnicity had a significant effect for several outcomes indicating lower QOL, self-management skills and higher depression for Chinese relative to non-Chinese irrespective of study arm: general health (*F* [1, 40]=4.00, *p* = .05), mental health (*F* [1, 40]=7.82, *p* = .01), cognitive functioning (*F* [1, 39]=5.95, *p =* .02), staff encouragement (*F* [1, 40]=9.61, *p =* .004), and kidney disease component score (*F* [1, 40]=7.13, *p* = .01), constructive attitudes (*F* [1, 40]=22.04, *p* < .001), and depression (*F* [1, 39]=13.50, *p* = .001). The effect of ethnicity on social functioning approached but did not reach significance in ITT analyses (*F* [1, 40]=3.88, *p* = .06).

Per protocol (PP) analyses indicated similar patterns of results for all outcomes with the exceptions that the effect of Chinese ethnicity for social functioning, shown as trend in ITT, was significant (*F* [1, 36]=4.87, *p* = .030). PP analyses confirmed that although scores in role limitations due to physical improved across sample over time, the improvement was significantly greater in C-DIRECT.

### Qualitative analysis

Thematic analysis has identified several key themes regarding the intervention’s acceptability and impact on patients’ motivation for behaviour change and self-management [59]. Intervention participants were positive about the program describing as useful and engaging and commenting positively on facilitators.

*They are here to encourage and help us, because they care for us and do not want us to suffer from complications. I think it is good. (R12).*

*We are old, and sometimes we forget, so for them (Facilitators) to remind us, it is a good thing. I like it. (R11).*

The useful elements were reinforcements through the regularly planned sessions, and having specific yet modest goals for weekly action. Some patients also felt motivated by the ‘personal agency’ approach that gave them choices over topics of sessions*.*

*She asked me to choose right? Want to go for exercise or diet or medication or the leg. So I choose foot care lah. Because, for medication I am used to it already. I take my medicines following the time. And then exercise, I do exercise too at home. So I told the nurse, I want to take care of my leg. I choose for foot care. I like that (choosing) (R34).*

*Diabetes has to be managed systematically and carefully, otherwise we are doomed. I know I have to do this – I like making my own plans and talk about what to do every week (R5).*

At the same time, participants highlighted some barriers that they encountered, such as language, or how failed attempts or lack of improvement in their health, affected their motivation to actualize what has been taught or to persist with behaviour change.

*They told me to change the amount of rice I eat, so I did. I tried repeatedly but the situation just did not change. I felt very frustrated and I wanted to give up. (R8).*

There were other criticisms and points of improvements. Patients felt that content of advice could be better personalised and tailored to their situation and needs. Some further added that they either heard or already knew the information shared in the sessions hence at times conversations were repetitive.

*Anyway I know how … So not so meaningful. Just like watching a movie… you watch it over and over again. So many nurses have told me before, so it is not so useful. (R14)*.

In general, nurses felt the intervention worked well, and found the training, and resources helpful. They noted that their communication skills were improved and interactions with patients seemed easer.

*Easier now to talk – before, sometimes patients pretend to be asleep when they see me, worry about lab results some they worry a lot… now they know this is for them to decide, they can tell me I can help them make goals. (RN 2).*

They did however note some difficulties. Facilitators were aware that they needed to use C-DIRECT approach more often (e.g. eliciting patients’ knowledge) but at times found it difficult to move away from the didactic manner and avoiding giving expert advice. They also commented that some patients (with many years post DM diagnosis) may already have learned to live with their symptoms, and thus were less interested in considering behavioural change.

*I see how better to discuss with patients not tell them what can or cannot. We did this in training but some don’t wanna change. Some say I know better I know my body, have diabetes for so long I know how. No problem lah. Then it is difficult, how to motivate them and set goals. I have to tell them to lower blood sugar cannot let this go. Cannot. (RN 1).*

## Discussion

Patients with DM and ESRD represent a high-risk group that are called to manage two chronic conditions and could benefit from self-management interventions. While health behaviour interventions are highly valued by HD patients [21], the time consuming nature of HD and scheduling considerations hinders participation and implementation. The C-DIRECT was developed to address this gap. To our knowledge, this is the first study to trial a chairside intervention specifically developed for patients with coexisting diabetes and end-stage renal disease. We aimed for C-DIRECT to be delivered in routine care by front care staff rather than highly specialized psychologists or other mental health professionals to enhance ‘in-house’ capacity and increase patient access and acceptability. Time requirements were purposefully kept at minimum to cater for both staff who are often under time duress and to allow implementation during HD session with due consideration for patients’ convenience.

We have shown that with appropriate training and support, C-DIRECT has the potential to be integrated into clinic practice with good recruitment and retention rate, attesting the feasibility and receptiveness of the program.

Qualitative interviews indicated that both patients and facilitators derived some benefits but there were challenges around content tailoring to patients’ needs and delivery/fidelity to non-didactic approach, which dominates health care encounters. The patients appreciated the encouragement and effort, as well as the regular contact and reminders by the facilitators. This perceived care and concern from nurses makes a big difference for the patients’ experience in health care [60]. Facilitators also showed value in improving their skills and communication approach. They appreciated the training and coaching and reported changes in skills and attitudes which they considered to have enhanced their practice beyond the scope of the study.

Analysis of clinical and patient reported outcomes indicated some benefits. For the clinical outcomes at follow-up, both groups showed improvements in HbA1c across the two assessments. The improvement in glycaemic control was noteworthy for C-DIRECT – HbA1c levels decreased by an average of 0.80% in C-DIRECT (from 9.57 to 8.78%) post intervention which is clinically relevant [61]. Analysis of data relative to clinical targets indicated that a significantly greater proportion of C-DIRECT had HbA1c levels below 8% at follow up relative to usual care. Moreover, these clinical gains were observed in patients who had so far not been successful in good glycaemic control (as indicated by their baseline values). The observed effects suggest that while UC comprising advice on diabetes care in a more traditional didactic approach can help to improve glycaemic control work, augmenting of UC with techniques from self-management and motivational interviewing may be even more beneficial. This is largely aligned with previous evidence previous evidence in the context of diabetes [62, 63].

The program was also shown to have important advantages for C-DIRECT patients in addition to the improvements in glycaemic control. The observed improvements in role limitations due to physical health suggest functional gains for C-DIRECT. The mechanisms whereby C-DIRECT, a multimorbid disease-specific intervention, produces functional benefits need to be explored further. Nonetheless, our preliminary data suggest that a brief clinic integrated intervention have some benefits for patients with coexisting diabetes and ESRD in QOL and clinical outcomes.

Interestingly, findings suggest variation in outcomes as function of ethnicity. Chinese patients reported worse QOL in several generic and disease specific domains, higher levels of depression and lower scores in constructive attitudes and approach relative to non-Chinese, in line with some prior work from Singapore with renal patients and in general population [35, 64]. While more work is needed to explore ethnic variations, our results underscore the need for future trials to consider ethnicity in the design, evaluation and implementation of interventions. Stratified sampling is strongly advised for future research. There is also need for a more nuanced understanding of patient needs in delivery of care. In terms of practice, health care providers need to be vigilant for poor adjustment among Chinese patients and consider culture-sensitive care and interventions.

While the observed improvements with this brief chairside intervention are certainly encouraging, they should be interpreted with caution as the study has several limitations. Firstly, this is an RCT with a small sample size and inclusion of multiple endpoints. This was because we aimed to explore preliminary effects and the feasibility of a bed/chairside, clinic-integrated intervention for DM ESRD patients – something that has not been done before. This trial is not sufficiently powered to draw any conclusions on efficacy as it allowed to capture only outcomes with high effect sizes with sufficient precision. The small sample also led to a slightly unbalanced randomisation with significantly more Chinese patients in the intervention arm. However, despite the low statistical power, this feasibility RCT served as a first step in demonstrating the feasibility of a subsequent, larger scale trial and can provide relevant input for larger sized studies.

Secondly, another potential weakness is the use of a bilingual interviewers for administration of study questionnaire when so requested/ preferred by patients. This has facilitated participation but may have fostered social desirability bias. Although the research staff remained blind to study arm allocation and was independent to patients’ renal care team and the C-DIRECT intervention facilitator, this may still have introduced some reporting bias into this subgroup of patients. CDIRECT was offered to those fluent in English or Mandarin – we had to exclude patients only fluent in Malay, Tamil, or other dialects due to logistical constraints. As however among the four official languages in Singapore (English, Mandarin, Malay, and Tamil), English is primarily used (83.1% of the population is literate in English) and literacy in at least two is the norm (73.2% is bilingual) [65], we believe that the program had reasonable reach in the target population.

Thirdly, self-selection bias cannot be ruled out as well**.** While the overall sample profile was representative of DM ESRD population, those who consented may have been more ready to change or concerned about health matters. This readiness is “*condition sine qua non”* for behaviour change and self-management.

Finally, interventionists and participants were not blind to treatment, which may introduce bias into the study. Hence it is suggested that future studies consider further blinding procedures.

## Conclusions

In conclusion, the trial demonstrated that this brief, clinic integrated intervention seemed to be feasible for the patient group and purpose studied.

The study protocol was found to be satisfactory in the eligibility, recruitment, and retention rates as well as the secondary outcome measure completion. Analyses of outcome measures indicated positive changes in QoL (role limitation due to physical health) and promising effect on HbA1c levels, with a significant increase in numbers of CDIRECT patients with HbA1c levels within clinical targets post CDIRECT.

Future studies are warranted to determine whether this brief, clinic-integrated intervention can have substantial benefits in the large, vulnerable, and growing population of people with coexisting Diabetes and ESRD. Of interest would be to explore the value of program for patients on home-based dialysis modalities such as peritoneal dialysis and the sustainability of (any) effects over time using long term follow up assessments.

In terms of practice, the results of this feasibility RCT suggest that nurses can be trained in brief psychological techniques and deliver self-management support for these high risk patients (DM-ESRD) during HD sessions. Due to time demands and schedule rigidity of dialysis routines for patients and staff alike, delivering interventions in time efficient manner is essential. Given this minimum investment of time for delivery, the CDIRECT program may of interest to dialysis front care who are at close and regular contact with patients.

## Abbreviations

CCI: Charlson Comorbidity Index
C-DIRECT: Combined Diabetes and Renal Control Trial
CKD: Chronic Kidney Disease
DDFQ: Dialysis Diet and Fluid Non-Adherence Questionnaire
DM: Diabetes Mellitus
DSES: Diabetes Self Efficacy Scale
ESRD: End-stage Renal Disease
HADS: Hospital Anxiety and Depression Scale
HD: Haemodialysis
HEIQ: Health Education Impact Questionnaire
HRQoL: Health-related Quality of Life
ITT: Intention to Treat
KDQoL: Kidney Disease Quality of Life Short Form
MCS: Mental Component Summary
MI: Motivational Interviewing
NKF: National Kidney Foundation of Singapore
NUS: National University of Singapore
PAID: Problem Areas in Diabetes Scale
PCS: Physical Component Summary
PP: Per Protocol
QOL: Quality of Life
RCT: Randomised Controlled Trial
SDSCA: Summary of Diabetes Self-Care Activities
SPSS: Statistical Package for the Social Sciences
UC: Usual Care
